# Strong evidence for ideomotor theory: Unwilled manifestation of the conceptual attribute in movement control

**DOI:** 10.3389/fpsyg.2023.1066839

**Published:** 2023-04-04

**Authors:** Yun Kyoung Shin, Seonggyu Choe, Oh-Sang Kwon

**Affiliations:** ^1^Department of General Education, University of Ulsan, Ulsan, Republic of Korea; ^2^Department of Biomedical Engineering, Ulsan National Institute of Science and Technology, Ulsan, Republic of Korea

**Keywords:** ideomotor theory, perceptual decision, affordance, voluntary action control, stimulus effect compatibility, the theory of event coding

## Abstract

Scientific understanding of how the mind generates bodily actions remains opaque. In the early 19th century, the ideomotor theory proposed that humans generate voluntary actions by imagining the sensory consequence of those actions, implying that the idea of an action’s consequence mediates between the intention to act and motor control. Despite its long history and theoretical importance, existing empirical evidence for the ideomotor theory is not strong enough to rule out alternative hypotheses. In this study, we devised a categorization-action task to evaluate ideomotor theory by testing whether an idea, distinguished from a stimulus, can modulate task-irrelevant movements. In Experiment 1, participants categorized a stimulus duration as long or short by pressing an assigned key. The results show that participants pressed the key longer when categorizing the stimulus as long than they did when characterizing it as short. In Experiment 2, we showed that the keypressing durations were not modulated by the decision category when the property of the decision category, the brightness of a stimulus, was not easily transferable to the action. In summary, our results suggest that while the perceived stimulus features have a marginal effect on response duration linearly, the decision category is the main factor affecting the response duration. Our results indicate that an abstract category attribute can strongly modulate action execution, constraining theoretical conjectures about the ideomotor account of how people voluntarily generate action.

## Introduction

Wittgenstein asked, “What is left over if I subtract the fact that my arm goes up from the fact that I raise my arm?” (Wittgenstein, 2010, §621). Understanding how the mind controls the body has been an elusive inquiry. Individuals can consciously access the intention to act and the perceived results of their actions. However, the mental process that connects the intention and the results occurs outside of awareness. For example, Libet (1985) found that readiness potentials indicating cerebral initiation precede the conscious intention to act. According to him, the causal arrangement of will and action is reconstructed in a retrospective manner. The discussion of whether a free-will over her action truly exists is a hard problem that might be beyond the scope of empirical research (Roskies, 2011). However, neuroscientific studies have shown that humans can at least consciously abort unconsciously initiated actions (Libet et al., 1993; Mirabella, 2007; Filevich et al., 2012; Mirabella, 2021), and the neural structure of the inhibitory process has been identified to some extent (Brass and Haggard, 2007). The ability to choose the best option by intentionally withholding potentiated action candidates, known as free choice, may allow for free will, rather than an omnipotent ability to do anything (Libet et al., 1983; Roskies, 2011). The philosophical viewpoint that opposes causal determinism supports free will by asserting that “*x* has control over *a* only if *x* has the ability to choose from an alternative course of action to act (McKenna and Justin Coates, 2004).

If the will is not a causal antecedent of initial action readiness, what does cause action? Affordance has been a powerful framework to account for the ways in which motor parameters are shaped concurrently with perception (Gibson, 1977). The perceived features of objects relevant to an action trigger cortical facilitation in the motor area (Chao and Martin, 2000; Grezes and Decety, 2002). This sensorimotor framework has been a prevailing paradigm for explaining the inextricable link between perception and action and avoiding the hard problem of mind–body dualism. Recent studies have debated whether the automaticity of affordance can be attenuated by cognitive control (Ellis and Tucker, 2000; Bub and Masson, 2010; Masson et al., 2011; Freeman et al., 2016 and Chong and Proctor, 2020). Influential theories that have emerged from those studies explain that the process by which one particular action is selected from among many alternatives triggered by environmental cues involves goal-driven decisions (Cisek and Kalaska, 2010; Cisek, 2012; Mirabella, 2014; Mirabella and Lebedev, 2017). Environments offer many action opportunities or demands, and the human brain continuously calculates the biomechanical ease of acting on an object (*cost*). That top-down process predicts an expected outcome based on the reward/risk (*value*) of the choice. Consensus between these multi-layered representations can induce an action that maximizes the total utility (*value-cost*) at a given moment. Both variables, the cost and the value, can bias a decision about what to do, but the motor parameters are prepared using only the cost. This process might help to adapt to a changing environment in real-time by picking up the best-fitted action quickly, while simultaneously preparing a set of action repertoires (Cisek and Kalaska, 2010; Cisek, 2012; Mirabella, 2014; Mirabella and Lebedev, 2017).

The affordance account might explain well how one movement is finally made to minimize the cost of achieving a desirable outcome. However, humans are not just beings who react passively to the features of a given environment; they are beings who can control their movements as they think. More than a century ago, ideomotor theorists suggested that the anticipation of an action’s sensory consequences leads to the generation of motor signals (Herbart, 1816; James, 1890). That theory was based on the conjecture that a bidirectional association builds up between motor signals and their sensory consequences through frequent coactivation (James, 1890). As such, the ideomotor theory proposes a seamless connection between idea and action, which implies that an idea alone can trigger action even without the conscious intention of an agent. William James once stated that “there were no room for any third intermediate principle of activity, like that called the will between the ideas themselves on the one hand and the conduct on the other” [*SIC*] (James and Skrupskelis, 1983, p.104). He explained that all fleeting ideas do not turn into motor signals because the default function of will from the “high center” is “to exert a constant inhibitive influence on the excitability of those below” (p. 103).

### The ideomotor phenomenon: Ouija board

As a cognitive antecedent to motor execution and adjustment, the ideomotor account sets an idea as a critical component of movement control. The ideomotor phenomenon indicates that movement is automatically controlled by a certain idea when the conscious intention is turned off. Carpenter (1852) attempted to demystify a variety of uncanny phenomena, such as dowsing rods, pendulums, and Ouija boards that the spiritualists considered to be supernatural, by explaining them as ideomotor phenomena. He explained ideomotor control as a complete subjection of the muscular power to the current dominant idea while the will is in abeyance. For instance, Ouija boards use a wheeled flat piece as an indicator to point to letters on the board. The mechanics of the wheeled indicator amplify tiny movements that the players initiate. Because the players do not experience a sense of agency in achieving the consequence, due to the absence of a conscious intention, they often misattribute the movement of the indicator to an external spiritual force. The ideomotor process is thus a sort of reflex in which the anticipation of a given result is the stimulus that directly and involuntarily prompts the muscular movements needed to produce it. The ideomotor pathway provides a clue about free will because the source for the agent’s action is reduced to a pure internal mental event. If a human action is triggered only by external events, such as a stimulus, a reward/punishment, or a mental representation related to those events, free will seems to have no place, even when a human actor has alternatives. When a hungry rat runs through a maze to get food, we cannot say the rat has free will, although the rat desires to do and choose one best candidate among others. The existence of an ideomotor pathway could provide a clue about self-deterministic behavior by opening the possibility that a mental state could be the causal antecedent of an action. The libertarian perspective on free will suggests that *x* has control over *a* only if *x* is the ultimate source of *a* (McKenna and Justin Coates, 2004). However, the contemporary ideomotor theory has deviated from its initial focus on the connection between an idea and movement and concentrates more on how actions are modulated by external events, such as a stimulus or an effect, presumably because a subject’s idea is neither observable nor easily controllable. In this study, we call those contemporary versions of ideomotor theories as perceptuo-motor and effecto-motor theories.

### A goal for action: The effecto-motor account as an ideomotor model variation

Most existing ideomotor studies have treated the action effect as the primary independent variable and demonstrated that effect events control action selection (Elsner and Hommel, 2001; Kunde, 2001a; Pfister et al., 2011; Pfister, 2019). Action selection is facilitated when a previously presented effect is the target stimulus (Elsner and Hommel, 2001) or when the effect (quiet or loud tone) is compatible with the property of the response (soft or forceful pressing) (Kunde, 2001a). Such studies deviate from the original theory and instead reflect the effecto-cognition explanation because they fail to specify how one particular movement is made (Kunde, 2001b; Shin and Proctor, 2012). The effect event intervenes in the early part of action preparation—selection—only under the instruction to achieve the effect (Shin and Proctor, 2012).

Evidence from the effecto-motor paradigm is not strong enough to exclude explanations other than ideomotor theory. For example, response facilitation might be due to the efficiency of compatible feedback, which can enhance general performance (e.g., Sharma et al., 2016). Major ideomotor theorists have proposed that an associative learning mechanism binding two events, an action and its effect, is the basic principle of the anticipatory process (Greenwald, 1970; Hommel et al., 2001; Eder et al., 2020; Sun et al., 2020). If that is so, a boundary condition is required to test the ideomotor mechanism and exclusively differentiate it from other general behavior theories.

### Stimulus to action: The perceptuo-motor account as an ideomotor model variation

Contemporary ideomotor theory, mostly the effecto-motor version, has developed to contrast itself with the stimulus-driven behavior model (Hommel et al., 2001). However, it also paradoxically accounts for perceptually induced action modulation. According to Prinz (2005), if “thinking of an act (or its effects) has the power to prompt and instigate that act, perceiving an action should instigate action.” Some literature assumes that the internal image created by anticipation has a quality similar to images triggered by sensory input, making obsolete the need for a bridging the state of an idea (Kunde, 2001a; Thomaschke, 2012). In that way, *ideomotor* has begun to be used interchangeably with the concept of perception-action coupling in the literature (Hommel et al., 2001; Bargh, 2005; Thill et al., 2013; Prinz, 2016). However, perceptually guided action has already been explained by affordance theory, as introduced earlier (Gibson, 1977). Affordance proposes a direct link between perception and action without requiring an internal stage that elicits an idea. Contemporary ideomotor theorists do not particularly distinguish whether a perception elicits a certain idea to induce action or directly induces the action. The stimulus–action coupling phenomenon is consistent with both ideomotor and affordance theories. Thus, behavioral data displaying perceptually induced action can neither distinguish those two theories nor provide evidence of the critical stage proposed by the ideomotor theory.

### Idea to action: The ideomotor model

Despite the long history and anecdotal evidence (Ouija, pendulum, and dowsing rod) of ideomotor control, empirical evidence demonstrating its most distinctive feature, automatic muscular activity driven by thought/idea/suggestion alone, using a rigorous experimental design has been lacking in the literature. Although contemporary ideomotor accounts have revived the original ideomotor theory, little evidence separates it from other theories, such as affordance, stimulus–response compatibility, associative learning, and performance feedback. As some have pointed out, the Theory of Event Coding is limited to explaining the late stages of perception and early cognitive antecedents of action; thus, it mostly explains response selection guided by a goal (Hommel et al., 2001; Shin et al., 2010).

The idea we suggest here is not a perceived stimulus or desirable effect but a highly abstract concept. As Hommel et al. (2001) suggested, event codes should be integrated by binding features that refer to both perceived features and sensory feedback from an action and that subserve both representational function and motor function. The symbolic code format allows overlap between features that refer to the same category, such as that an apple and a ball are both round (Prochazkova and Hommel, 2020). We suggest that the representational code for ideomotor control is a highly abstract, amodal code that is not restricted by the sensory mode in which subjects first perceive an impression but directly coordinates muscular activity in one go. This symbolic code seems to require many more steps to generate action because this mental event does not resemble the physical event or muscle control required to generate causal relevance. That is why Thorndike (1913) fervently criticized it as magical superstition to set up the ideomotor link “to produce the act which it represents or resembles or is ‘an idea of,’ or ‘has as its object.’”

The best way to reveal the function of ideomotor control is to observe what it would look like if the ideomotor route was hampered (Wheaton and Hallett, 2007; Weiss et al., 2008). The symptoms are highly varied (Sirigu et al., 1995; Buxbaum et al., 2003), but researchers have found that the primary failure of ideomotor apraxia occurs in executing the proper motor schema into concrete movement while motor function and perception are comparably intact (De Renzi et al., 1980; Goldenberg, 2003).

**Figure 1:**
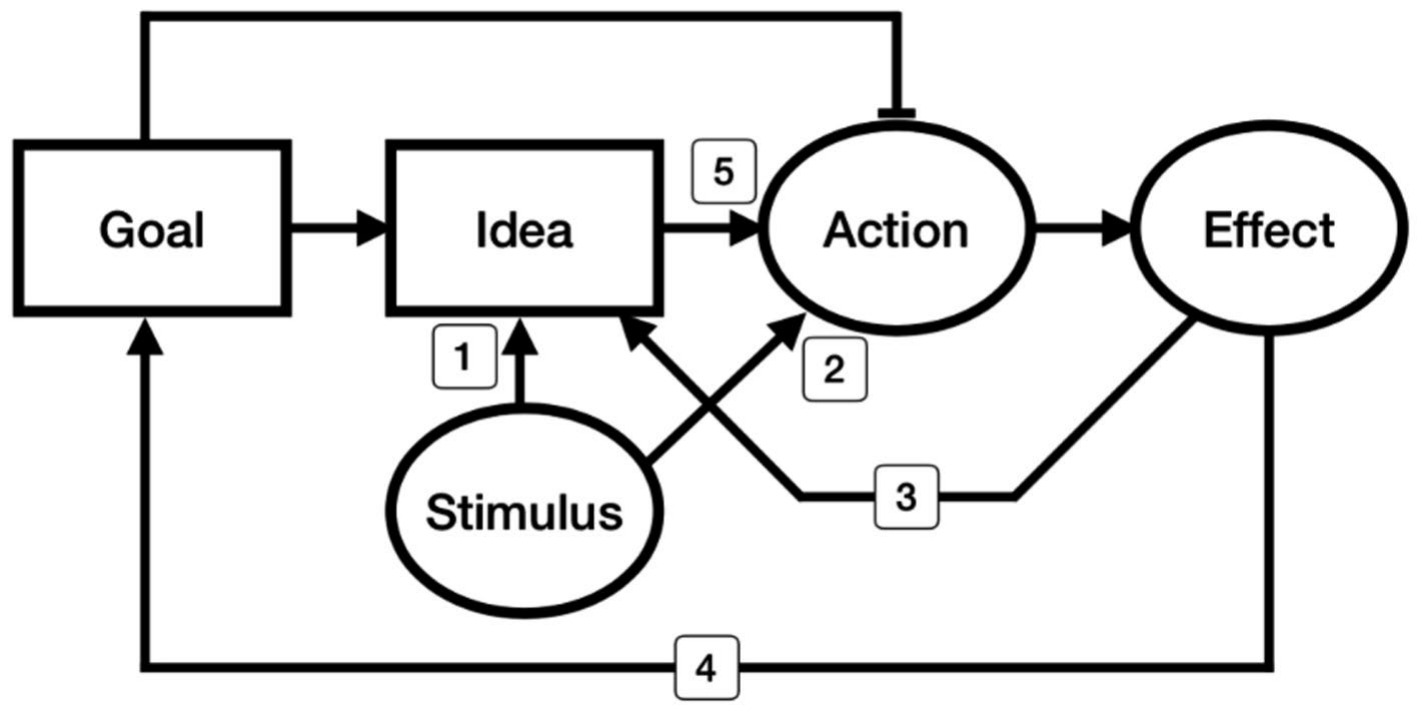
Schematic illustration of current ideomotor accounts. Each circle represents an observable event, stimulus, action, or effect. Each rectangle represents an unobservable process, goal, or idea. Arrows indicate the excitatory process, and a flat bar is an inhibitory process. Routes 1 and 2 represent the perceptuo-motor accounts: Route 1 is the induction of an idea related to an action’s sensory consequence *via* the perceived property of the stimulus. Route 2 is stimulus-guided action without an intermediate internal stage; thus, the action is selected based on biomechanical ease while adjusting control *via* visual guidance, similar to affordance. Routes 3 and 4 represent the effecto-motor accounts: Route 3 involves the anticipation of a sensory consequence, and Route 4 is goal-related instrumental action selection. Route 5 represents the ideomotor route, which is investigated in this study and was William James’s original hypothesis: A decision property (idea) automatically builds up a motor command even when it is not task-relevant.

We classified existing phenomena related to variations of ideomotor theory into five categories, schematically illustrated as five routes in Figure 1. The perceptuo-motor account emphasizes activation routes 1 and 2. Route 1 is described by Prinz (2005): Perceiving a stimulus property induces an idea of sensory consequence. Route 2, which activates motor patterns without the mediation of an internal state of an idea, is affordance. The effecto-motor account considers routes 3 and 4. Because the effect event itself, which occurs after the movement, cannot directly modulate the movement, the effect “image” should be called upon during action preparation. If the effect image is represented as a goal state, the action becomes instrumental in bringing about an intended outcome, which is route 4. For example, Knuf et al. (2001) observed that a perceived scene in which a moving ball missed its target position induced bodily movement in the direction needed to modify the ball’s trajectory. Route 3 indicates the anticipatory process of sensory consequence (Kunde, 2001a). The two perceptuo-motor routes (routes 1 and 2) and the two effecto-motor routes (routes 3 and 4) have rarely been distinguished within the ideomotor literature. With a few exceptions (c.f., Knuf et al., 2001), most studies have treated the perceptuo- and effecto-motor routes as if they were the same, without specifying whether the effect image or the perceived image is the leading cause controlling an action. Route 5 is the excitatory pathway from conceptual attributes to action, which is the key component of ideomotor theory. The perceptuo- and effecto-motor accounts implicitly assume this route 5 connection when they suggest that a stimulus or effect induces a related idea (routes 1 and 3) that leads to motor activation (route 5). For this study, we developed an experimental paradigm to reveal a true ideomotor phenomenon, the subconscious manifestation of an idea through muscular execution. We adopted a perceptual categorization task to manipulate the attributes that participants were focusing on. Specifically, we asked participants to categorize stimulus durations as long or short by pressing an assigned left or right key, and we observed how their decision category was transmitted to the duration of each keypress. We expected that the representational code of judging whether a stimulus was long or short would also generate movement in pressing the key for a long or short time. This categorization task can sensitively discern the predictions of the idea-driven ideomotor theory from other explanations, such as the perceptuo- or effecto-motor accounts. Both perceptuo-motor and ideomotor accounts expect the stimulus duration to modulate the response duration. However, ideomotor theory predicts that the modulation will strongly reflect the result of the categorizational attribute (idea). In contrast, the perceptuo-motor theory predicts that the modulation will reflect the stimulus property as-is. The effecto-motor version cannot explain why the decision category generates the corresponding movement when it is not required to do so.

## Experiment 1

We designed a categorization-action paradigm to examine how participants’ categorical decisions would modulate their task-irrelevant response duration. In the simplest version of the task, participants were required to categorize audiovisual stimuli with two durations, long and short, by pressing the left or right key (Experiment 1A). In Experiment 1B, we examined whether the keypressing time modulation that we found in Experiment 1A reflected the decision category or the stimulus feature by varying the stimulus into six different durations and requiring the same binary decision.

### Method

#### Participants

Twenty-two students from the Ulsan National Institute of Science and Technology who were naive to the purpose of the study participated in Experiments 1A and 1B sequentially on the same day. The sample size was determined using the sample sizes of existing studies that adopted choice response tasks with keypresses to answer-related research questions (8 participants, Kornblum and Lee, 1995; 17 participants, Eimer, 1995; 24 participants, Hommel, 1997; 40 participants, Hommel, 1996; mean of four studies: 22). The Institutional Review Board of the Ulsan National Institute of Science and Technology approved this study (UNISTIRB-18-39-C). All methods were performed in accordance with the relevant guidelines and regulations. All participants signed a written informed consent form before the experiment and received a monetary reward regardless of their task performance. All had normal or corrected-to-normal vision and normal hearing. One participant, who registered the same response in all trials, was excluded from the analyses.

#### Apparatus and stimuli

The stimuli were generated using MATLAB and the Psychophysics Toolbox (Pelli, 1997) on a 15-inch MacBook Pro laptop computer (Apple, Cupertino, CA, USA). The target stimulus was a 1 kHz pure tone concurrently delivered with a dark gray square (2.3 cm x 2.3 cm) at the center of the screen. The duration of this audiovisual target presentation was 100 or 150 ms in Experiment 1A and 85, 100, 115, 135, 150, or 165 ms in Experiment 1B. In Experiment 1B, the stimuli with three shorter durations (85, 100, 115 ms) were considered short stimuli, and the stimuli with three longer durations (135, 150, and 165 ms) were considered long stimuli for the binary decision.

#### Procedure

**Figure 2:**
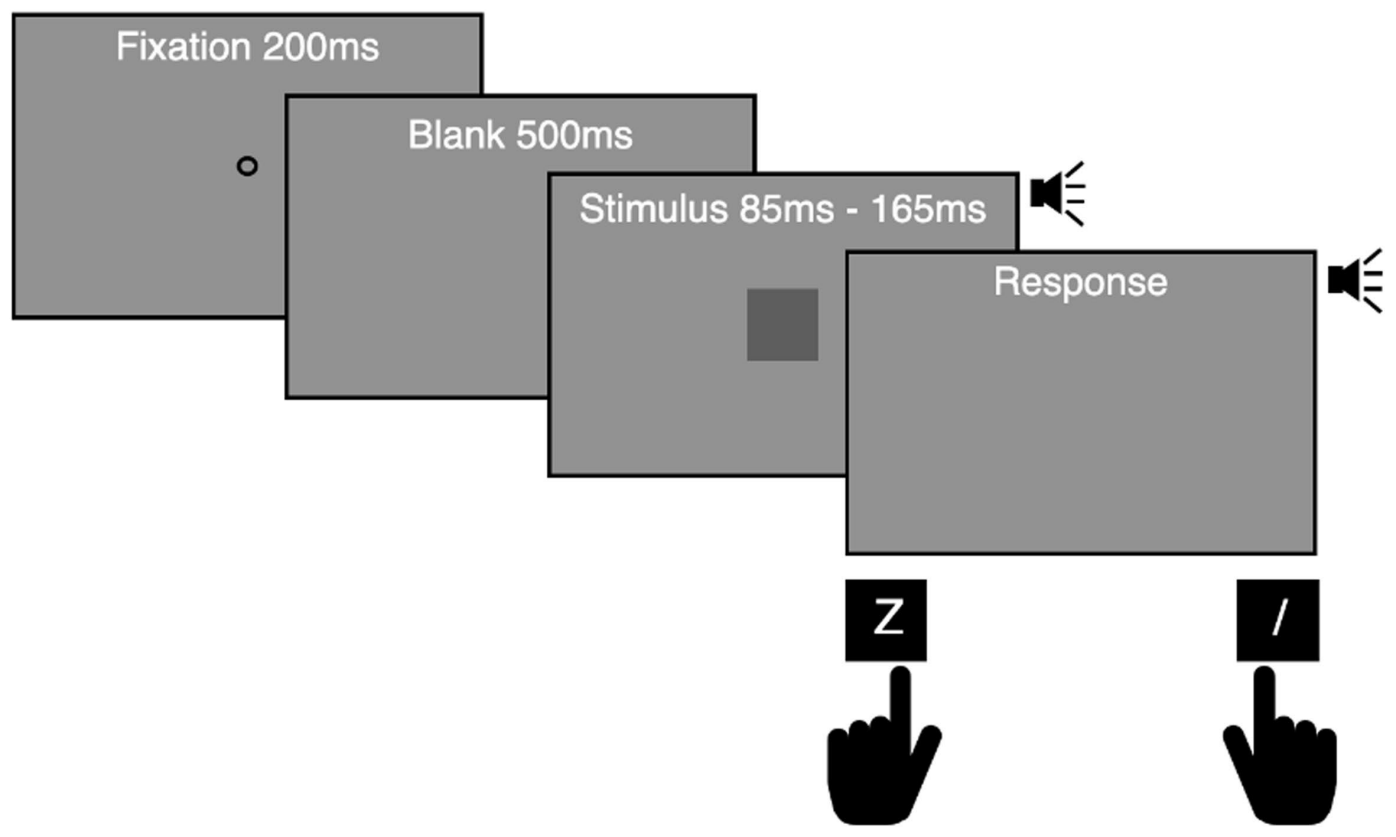
Experimental procedure used in Experiments 1A and 1B. Participants were asked to report whether the duration of an audiovisual stimulus was long or short by pressing a left (Z) or right (/) key, which was counterbalanced across participants. A 1 kHz pure tone was presented while the correct key was pressed.

Participants were asked to indicate whether they thought the audiovisual stimulus was long or short by pressing the “Z” or “/” key with their left or right index finger. They were instructed to respond as accurately and speedily as possible after stimulus termination (Figure 2). Mapping between the duration of the stimuli and the correct key was counterbalanced across participants. Participants were not given instructions on how long to press the key and were unaware that the keypress time was recorded for data collection. Participants pressed any key to start a block after instructions, and a fixation point was presented for 200 ms. After 500 ms of a blank gray screen, a target stimulus was presented. The duration of target stimuli varied randomly across trials. After a 1-s inter-trial interval, the fixation point for the subsequent trial appeared. When a participant pressed the correct key, a 1 kHz pure tone sounded while the key was being pressed. When a participant made a mistake, such as an early response before stimulus termination or incorrect categorization, a visual feedback message (“Too early!” or “Error!”) was given for 700 ms. One hundred trials per duration (200 trials in total) were conducted in Experiment 1A, and 36 trials per duration (216 trials in total) were conducted in Experiment 1B.

### Results

#### Experiment 1A: Binary decision task with two stimulus durations

Reaction times (RTs), measured as the interval between the onset of the stimulus and the response (RTonset), did not differ by stimulus duration, *t*(20) = 1.117, Cohen’s *d* = 0.244, *p* = 0.277. However, the interval between the offset of the stimulus and the onset of the response (RToffset) was significantly longer for the short stimuli: RToffset short: 407 ms, RToffset long: 367 ms, *t*(20) = 4.79, Cohen’s *d* = 1.046, *p* = 1.10e-4. This 41 ms difference was comparable to the stimulus duration difference (50 ms). These results suggest that the decision-making process is anchored to the stimulus onset rather than the stimulus offset. Therefore, in the subsequent experiments, only RTonset was analyzed. The mean accuracy was 89% in the short stimulus condition and 81% in the long stimulus condition, *t*(20) = 2.534, Cohen’s *d* = 0.553, *p* = 0.019.

**Figure 3:**
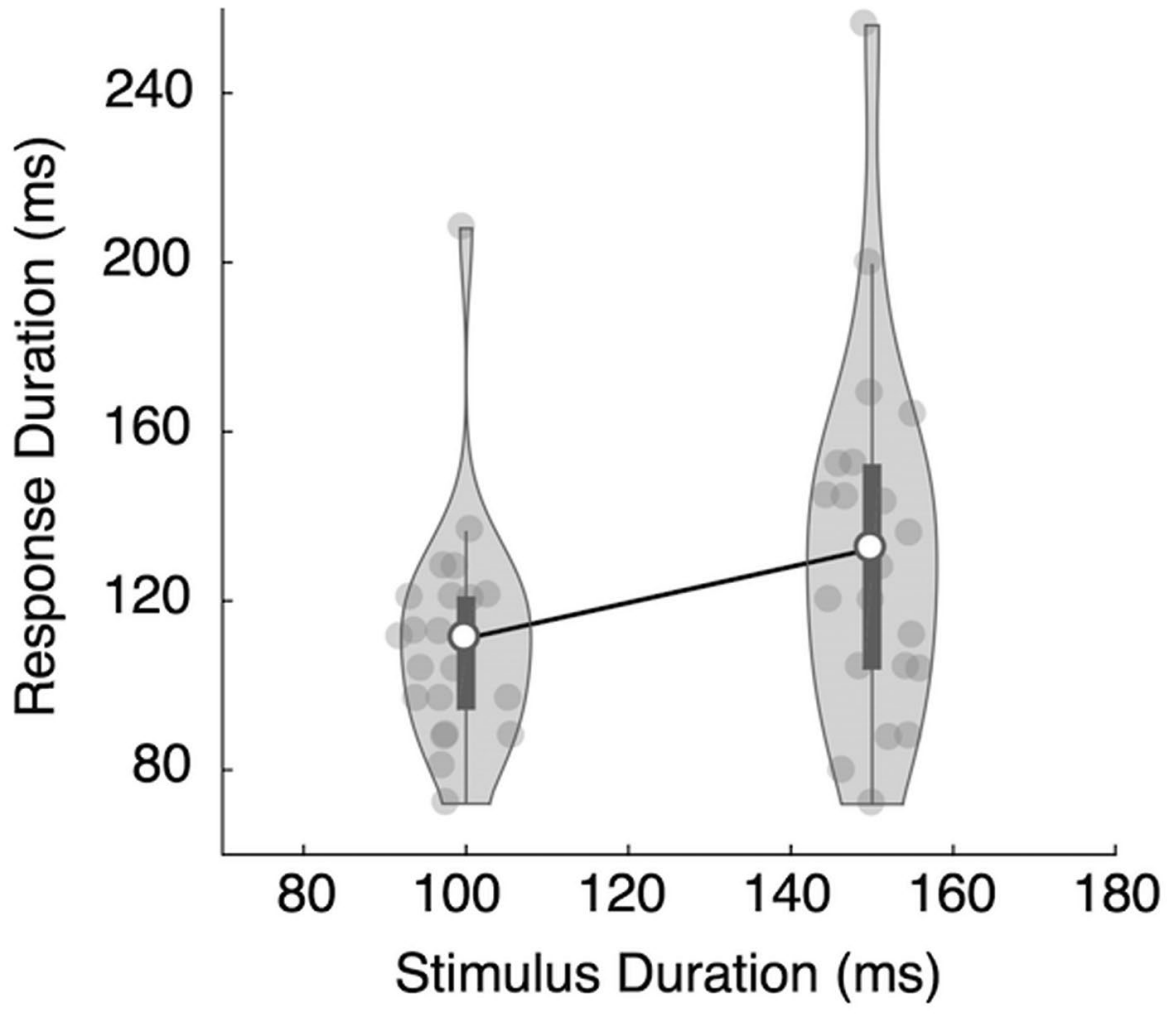
Results of Experiment 1A. The mean response duration for the long stimulus (150 ms) was longer than the mean response duration for the short stimulus (100 ms), and the difference was statistically significant. The dark bars represent the first and the third quartiles.

More importantly, the duration of the keypress (response duration or RD) was longer when correctly categorizing a stimulus as long and shorter when correctly categorizing it as short: RDshort = 111 ms vs. RDlong = 132 ms, *t*(20) = 3.638, Cohen’s *d* = 0.794, *p* = 0.0016 (Figure 3). During incorrect trials, the response durations tended to be longer for the short stimuli than for the long stimuli (RDshort = 131 ms, RDlong = 113 ms), though this difference was not statistically significant, *t*(20) = 1.679, Cohen’s *d* = 0.366, *p* = 0.109.

#### Experiment 1B: Binary decision task with six stimulus durations

**Figure 4:**
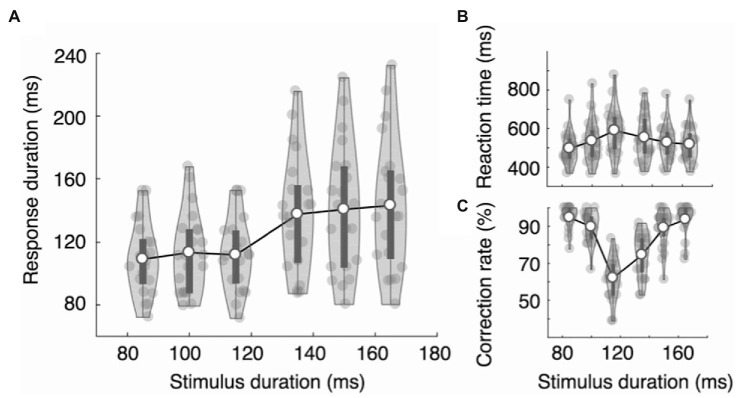
Results of Experiment 1B. **(A)** The response durations showed categorical patterns. The effect of stimulus duration was statistically significant, and the RD increase ratio was significantly larger between categories than within categories, demonstrating categorical RD modulations. Reaction time (from stimulus onset to response onset) increased **(B)** and mean accuracy decreased **(C)** as the stimulus duration approached the decision boundary. The dark bars represent the first and the third quartiles.

In Experiment 1B, participants had to categorize six stimulus durations as short or long and report by pressing the left or right key. The main question was whether the RDs would increase linearly, reflecting the stimulus durations, or be clustered into two groups, reflecting the decision categories. The results show that the RDs did not linearly increase as a function of the stimulus duration but were clustered into two separate categories: RDshort = 109, 114, and 112 ms vs. RDlong = 138, 141, and 144 ms, *F* (5,100) = 19.82, ηp^2^ = 0.498, *p* = 1.11e-13 (Figure 4A). Tukey’s test showed that the RDs in the three short stimulus conditions did not statistically differ from one another (*p* > 0.96), nor did those in the three long stimulus conditions (*p* > 0.88). However, all pairs between short and long conditions differed significantly (*p* < 0.0001). To further quantify the categorical pattern of RDs, we examined the RD increase ratio, which we defined as the ratio between the increase in RD and the increase in stimulus duration (RD increase/stimulus duration increase). We used the RD increase ratio rather than the RD increase itself because the stimulus duration increased by 20 ms between categories, whereas it increased by 15 ms within each category. We used six stimulus duration conditions (85, 100, 115, 135, 150, and 165 ms), and the means of the RD increase ratio for the five duration increase steps, 85 to 100 ms, 100 to 115 ms, 115 ms to 135 ms, and 135 to 150 ms, were 0.282, −0.098, 1.295, 0.202, and 0.172, respectively. The RD increase ratio was thus highest between categories. The results of repeated measure ANOVA show that the difference was statistically significant, *F* (4,80) = 12.72, ηp^2^ = 0.389, *p* = 4.67e-08. Tukey’s test showed that the RD increase ratio between categories (115 to 135 ms) differed significantly from the other four RD increase ratios (*p* < 0.0001), and none of the other pairs showed a statistically significant difference (*p* > 0.39).

Although those analyses show that the RDs were mainly modulated by the category effect, ANOVA is not an ideal test to depict the linear characteristics of a data set. Therefore, we developed a model that captures the linear stimulus effect and the category effect at the same time. Specifically, we fit a linear model with a category effect parameter to the data.


(1) $$RD=a*\mathrm{stimulus}\;\mathrm{duration}+b+c_{\mathrm{long}\;\mathrm{category}}$$

where *a* represents the slope of the overall linear effect; *c_long category_*, which applies only to long category responses, represents the category effect; and *b* represents the intercept. Those results show that the slope (*a*) and category effect (*c_long category_*) were significantly larger than zero (slope: mean = 0.14, *t*(20) = 2.91, Cohen’s *d* = 0.63, *p* = 0.009; category effect: mean = 22.2, *t*(20) = 4.12, Cohen’s *d* = 0.90, *p* = 0.0005). Note that the expected increase in RD due to the linear effect is only 2.8 ms at the category boundary (i.e., between 115 and 135 ms). Taken together, our results show that the RD was modulated by both the stimulus duration and decision category.

RTonset showed an inverted V-shape (RTs = 496, 535, 590, 553, 527, and 518 ms, Figure 4B), and the correct response rates showed a V-shaped function across stimulus durations (mean accuracy = 95, 90, 62, 75, 89, and 94%, Figure 4C). The RTonset of correct trials differed significantly across stimulus durations, *F*(5,100) = 16.45, ηp^2^ = 0.451, *p* = 8.03e-12, being longest (590 ms) when the stimulus duration was 115 ms and shortest (496 ms) when the stimulus duration was 85 ms (Figure 4B). However, the mean RTonset of the three long presses was not longer than that of the three short presses: RTlong = 533 ms, RTshort = 541 ms, *t*(20) = 1.090, Cohen’s *d* = 0.238, *p* = 0.289. The mean correct response rate was 84%, and it differed significantly across stimulus durations, *F*(5,100) = 72.39, ηp^2^ = 0.783, *p* = 2e-16, ranging from 62% (115 ms stimuli) to 95% (85 ms stimuli) (Figure 4C).

Among the six duration conditions, all subjects made at least one incorrect response in the 115 ms and 135 ms conditions. The mean error rate was 38% for the 115 ms condition and 25% for the 135 ms condition. The RDs of the incorrect trials were significantly longer in the 115 ms condition than in the 135 ms condition: RD115 = 135 ms, RD135 = 115 ms, *t*(20) = 3.544, Cohen’s *d* = 0.773, *p* = 0.002. In other words, participants pressed longer when they miscategorized a short stimulus than when they miscategorized a long one. The RTs and error rates gradually increased as the stimulus durations approached the category boundary (Figures 4B,C), suggesting that the adjustments in RD are not likely due to the categorical perception. The participants perceived the stimuli near the category boundary to be more similar than those far from the boundary; however, the perceived similarity was not reflected in the RDs. They did not press a key longer when they perceived it to be long but when they categorized it to be long.

### Experiment 1 discussion

Participants were required to press the left or right key to indicate whether a presented stimulus was long or short. In previous behavioral experiments, keypressing RDs have not often been the main concern because RTs sensitively measure selection cost or task difficulty, but recently this subtle measurement has received attention despite the small amount of variance to test (Pfister et al., 2022). Although the participants were not instructed about how long they should press the key, the task-irrelevant RDs were modulated by the nominal category participants used to decide what key to press. The RD was adjusted simply when two stimulus lengths (100 vs. 150 ms) were presented (Experiment 1A), but we could not exclude the possibility that the perceived stimulus adjusted the RD. Therefore, we presented six different stimulus lengths in Experiment 1B and required participants to categorize those six lengths into two categories. The RDs in Experiment 1B clearly show a dichotomous pattern reflecting the categories they used to decide, as ideomotor theory predicts, rather than a continuous pattern. Some could argue that the results of Experiment 1B might reflect that the participants perceived stimuli within the same category as being equivalent, but the participants made more errors in distinguishing between the middle two stimulus lengths, indicating that they perceived the six lengths differently. A simple linear model was unable to fit the RD, but a linear model with a category effect parameter explained the RD and showed that perceived stimuli linearly adjusted the RD. As shown in Figure 1, the perceived feature can still impact action through route 1 and ideomotor route 5, allowing the ideomotor account to parsimoniously explain both minor adjustments caused by stimuli and major adjustments due to the decision category.

## Experiment 2

In Experiment 2A, we replicated Experiment 1B. In Experiment 2B, we examined the effect of the task-irrelevant stimulus feature of duration on RD when the target feature for binary categorization was the luminance of the target.

### Method

#### Participants

We recruited a group of 40 students who did not participate in Experiment 1 from the Ulsan National Institute of Science and Technology. All participants were naive to the purpose of the study. They signed an informed consent form, and the study was approved by the Institutional Review Board of the Ulsan National Institute of Science and Technology. All subjects were financially compensated for their participation, and all methods were performed in accordance with the relevant guidelines and regulations.

#### Apparatus and stimuli

**Figure 5:**
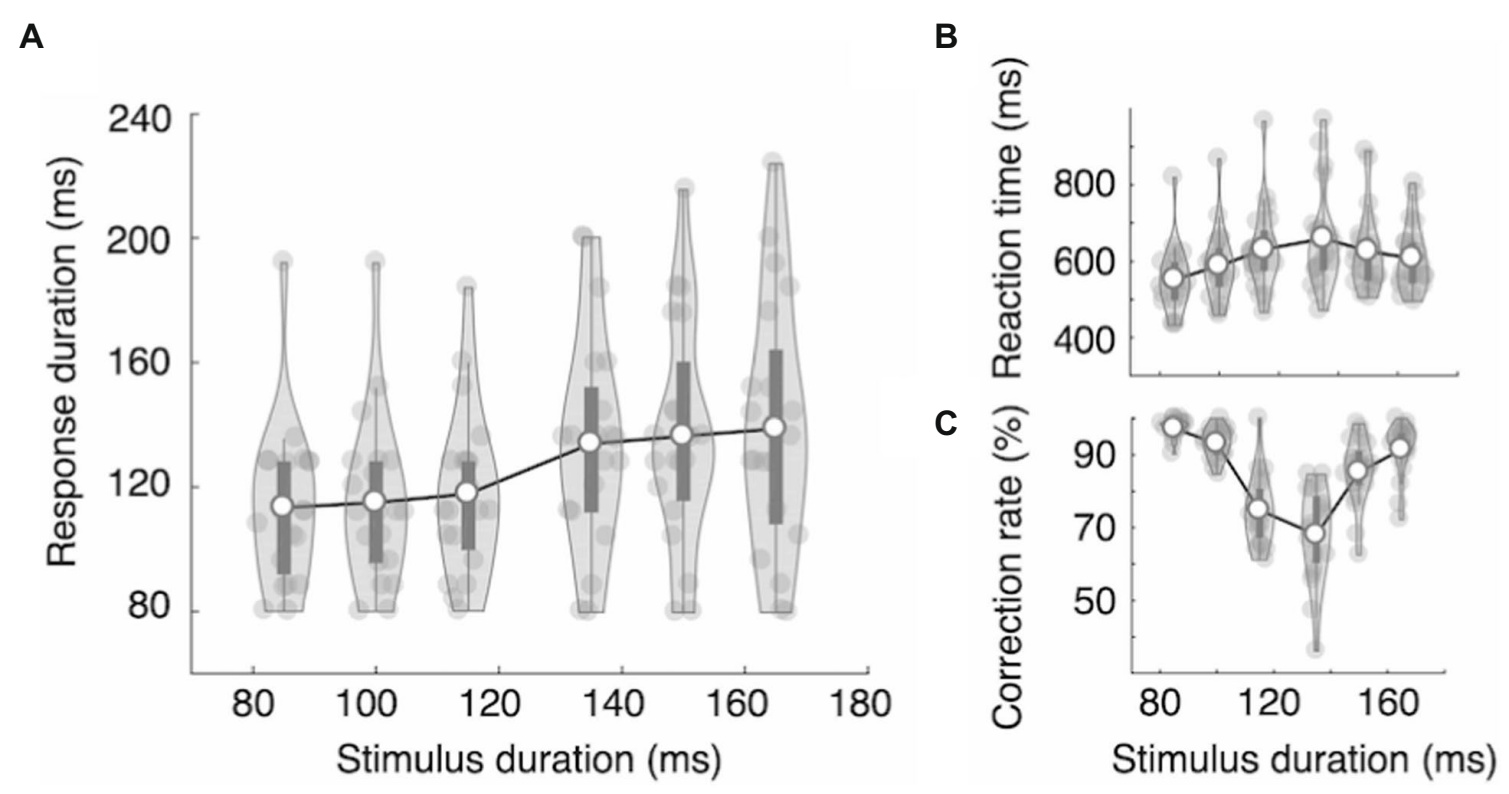
Results of Experiments 2A. In Experiment 2A, we repeated the procedure of Experiment 1B in a different experimental environment. Both Experiment 2A and 2B were conducted in the same environment. The results largely replicated Experiment 1B. **(A)** The response durations displayed categorical patterns, with a significantly larger increase ratio between categories. As the stimuli approached the decision boundary, reaction time increased **(B)** and the mean accuracy decreased **(C)**. The dark bars indicate the first and third quartiles.

**Figure 6:**
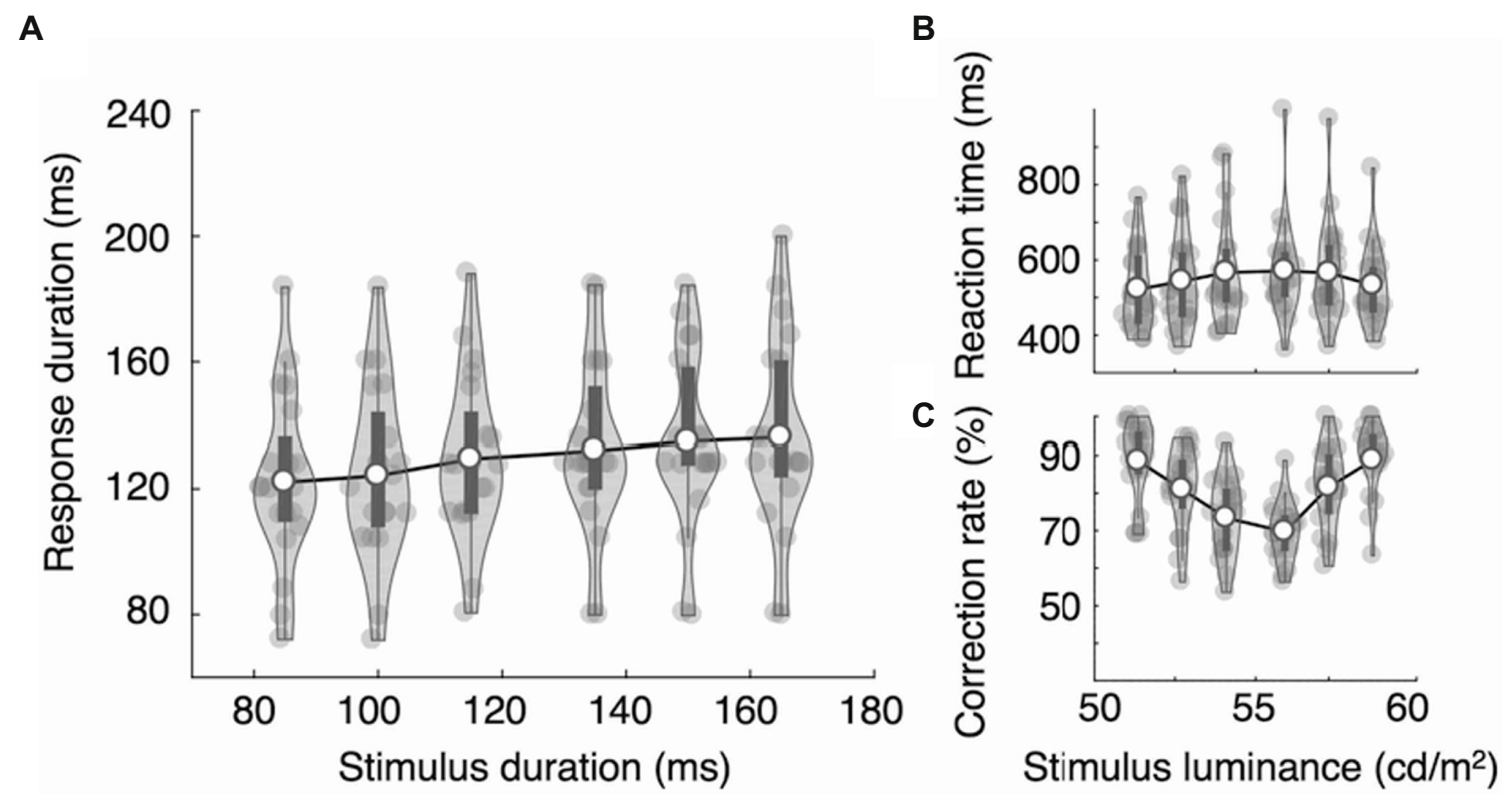
Results of Experiments 2B. Participants were asked to press the right (or left) key to categorize three bright stimuli and the left (or right) key to indicate the three dark stimuli in the luminance decision task. **(A)** Response durations increased linearly in the luminance decision task. Reaction time increased **(B)** and the mean accuracy decreased **(C)** as the stimuli approached the decision boundary. The dark bars indicate the first and third quartiles.

The stimuli were presented using a DLP projector (PROPixx, VPixx Technologies. Inc., Saint-Bruno, QC, Canada; 1920 H × 1,080 V; 120 Hz refresh rate; linear gamma) on a screen placed 130 cm in front of each participant. The Psychophysics Toolbox (Brainard and Vision, 1997; Pelli, 1997), in conjunction with MATLAB (Natick, MA, USA), was used for stimulus presentation and response collection. The visual stimulus, a gray square that appeared in the center of the screen, was accompanied by a 1 kHz pure tone in both Experiments 2A and 2B. The side length of the square stimulus was 3.45 degrees in visual angle. The procedures in Experiment 2A were identical to those in Experiment 1B. The duration of the audiovisual stimulus presentations varied (85, 100, 115, 135, 150, or 165 ms), and luminance was fixed (33.87 cd/m2). In Experiment 2B, six different luminance levels (51.1, 52.6, 53.6, 56.1, 57.9, and 58.9 cd/m2) were presented as a feature of the square stimulus. The luminance levels were selected to roughly match the decision difficulty of the duration decision task in Experiment 2A (Figures 5C, 6C), based on the results of a pilot test. The duration distribution was the same as in Experiment 2A (85, 100, 115, 135, 150, and 165 ms). The combination of six luminance levels and six durations made 36 stimulus types. Each stimulus type was presented 12 times, totaling 432 trials.

#### Procedure

Twenty participants performed the duration decision task (Experiment 2A), and the other 20 participants performed the luminance decision task (Experiment 2B). Participants were randomly assigned to one of the two tasks. The duration decision task was identical to Experiment 1B, except for the laboratory environment and the experimenter. In the luminance decision task, participants were asked to report whether the visual component of the stimulus was dim or bright by pressing the “Z” or “/” key with the left or right index finger, respectively. In both the duration and luminance tasks, participants were given no instructions about how long to press the key, and they were not aware that the pressing time was recorded for data collection.

### Results

#### Experiment 2A: Binary decision task with six stimulus durations

The results of Experiment 2A largely replicate the results of Experiment 1B. The RTs showed an inverted V-shape as a function of stimulus duration (RTonset = 552, 589, 631, 659, 626, and 608 ms; Figure 5B), and the differences were statistically significant, *F*(5,95) = 12.81, ηp^2^ = 0.403, *p* = 1.61e-9. The mean RTonset of the three long durations (631 ms) was significantly longer than that of the three short durations (591 ms), *t*(19) = 2.533, Cohen’s *d* = 0.566, *p* = 0.020. The mean accuracy showed a V-shape as a function of stimulus duration, and the differences were statistically significant: mean accuracy = 97, 93, 75, 68, 85, and 92%, *F*(5,95) = 46.48, ηp^2^ = 0.710, *p* < 2e-16.

More importantly, we found the same strong effect of decision category on RD as we found in Experiment 1B. The RDs tended to be either on the short side (RDshort = 113 ms, 114 ms, 118 ms) or the long side (RDlong = 134 ms, 136 ms, 139 ms). RD differences across stimulus durations were statistically significant, *F*(5,95) = 13.93, ηp^2^ = 0.423, *p* = 3.30e-10 (Figure 5A). Tukey’s test showed that RDs in the three short stimulus conditions did not differ statistically among themselves (*p* > 0.91), nor did those in the three long stimulus conditions (*p* > 0.89). However, all pairs between short and long conditions differed significantly (*p* < 0.006). We again examined the RD increase ratio. The means of the RD increase ratio for the five duration increase steps, 85 to 100 ms, 100 to 115 ms, 115 to 135 ms, and 135 to 150 ms, were 0.108, 0.182, 0.807, 0.164, and 0.157, respectively. As expected, the RD increase ratio was highest between categories (i.e., 115 to 135 ms). The results of repeated measure ANOVA showed that the difference was statistically significant, *F*(4,76) = 4.94, ηp^2^ = 0.206, *p* = 0.001. Tukey’s test showed that the RD increase ratio between categories (115 ms to 135 ms) was significantly different from all other RD increase ratios (*p* < 0.012), and none of the other pairs showed statistically significant differences (*p* > 0.99).

We also applied the linear model with a category effect parameter (Eq. 1) to capture the linear stimulus effect and category effect concurrently. Those results show that the slope (*a*) and category effect (*c_long category_*) were significantly larger than zero (slope: mean = 0.15, *t*(19) = 2.75, Cohen’s *d* = 0.62, *p* = 0.013; category effect: mean = 13.3, *t*(19) = 2.89, Cohen’s *d* = 0.65, *p* = 0.009). Note that the expected RD increase due to the linear effect is only 3.0 ms at the category boundary (i.e., between 115 and 135 ms). Taken together, our results show that the RD was modulated by both stimulus duration and decision category.

#### Experiment 2B: Binary decision task with six levels of stimulus luminance

Participants were asked to categorize whether the stimulus was bright or dim and to report by pressing a left or right key. The stimuli varied across six levels of brightness, and the stimulus durations also varied in six levels but were irrelevant to the categorization decision. Reaction times showed an inverted V-shape as a function of the stimulus luminance (RTonsets = 522, 543, 567, 571, 565, and 533 ms; Figure 6B). The effect of the stimulus luminance on reaction times was statistically significant, *F*(5,95) = 3.50, ηp^2^ = 0.156, *p* = 0.006. However, the mean RTonset of the bright stimuli did not differ significantly from that of the dim stimuli (RTbright = 556 ms and RTdim = 544 ms), *t*(19) = 0.770, Cohen’s *d* = 0.172, *p* = 0.451. The mean accuracy (87, 80, 72, 69, 80, and 88%; Figure 6C) showed a statistically significant V-shape as a function of the stimulus luminance, *F*(5,95) = 25.67, ηp^2^ = 0.575, *p* = 2.65e-16. Reaction times and mean accuracy did not differ significantly as a function of stimulus duration: RTs = 544, 543, 539, 544, 559, and 544 ms, *F*(5,95) = 1.18, ηp^2^ = 0.058, *p* = 0.327; mean accuracy = 78, 80, 78, 80, 80, and 80%, *F*(5,95) = 1.15, ηp^2^ = 0.057, *p* = 0.339.

Next, we examined the RDs applying two-way repeated measures ANOVA. The results show that the RDs did not vary systematically across the luminance levels, *F*(5,95) = 0.19, ηp^2^ = 0.010, *p* = 0.966, which rules out the possibility that the pattern of RDs observed in Experiments 1B and 2A is related to the reaction time or accuracy in any manner. The RDs were modulated by the task-irrelevant stimulus durations *F*(5,95) = 11.12, ηp^2^ = 0.369, *p* = 1.89e-08. However, unlike in the duration decision task, the RDs did not show a categorical pattern but instead increased linearly as the stimulus durations increased (RDs = 122, 124, 130, 132, 135, and 136 ms; Figure 6A). Tukey’s test showed a significant difference between stimulus duration conditions (85 ms vs. 115, 135, 150 ms, and 100 ms vs. 135, 150, 165 ms; *p* < 0.05). However, there was no distinguished cluster resembling the results of the duration decision task. Notably, RD at a stimulus duration of 115 ms did not differ significantly from RD at 135 ms (*p* = 0.905), which contrasts with the significant differences between those durations in Experiments 1B and 2A, when they represented category boundaries. The interaction between the luminance level and stimulus duration was not statistically significant, *F*(25,475) = 1.225, ηp^2^ = 0.061, *p* = 0.21. When we examined the RD increase ratio, we found that the means for the five duration increase steps, 85 to 100 ms, 100 to 115 ms, 115 to 135 ms, and 135 to 150 ms, were 0.138, 0.353, 0.127, 0.180, and 0.085, respectively. The results of repeated measure ANOVA did not show a statistically significant difference between steps, *F*(4,76) = 1.35, ηp^2^ = 0.066, *p* = 0.259.

We again applied the linear model with a category effect parameter (Eq. 1). Those results show that the slope (*a*) was significantly larger than zero (slope: mean = 0.19, *t*(19) = 3.53, Cohen’s *d* = 0.79, *p* = 0.002). However, the category effect (*c_long category_*) was not significantly different from zero (category effect: mean = −0.47, *t*(19) = −0.27, Cohen’s *d* = −0.06, *p* = 0.792). Unlike in the duration judgment task (Experiments 1B and 2A), the response duration was modulated only by the stimulus duration.

### Experiment 2 discussion

In Experiment 2A, we replicated the Experiment 1B results using the same methods but different experimenters, devices, and room conditions. We also tested whether a task-irrelevant change in the stimulus duration, which was a task-relevant feature in Experiment 2A, modulated the motor properties of participants categorizing six luminance levels in a binary fashion. We have not yet confirmed whether the brightness decision controls the action in some other way, such as generating a more forceful action for the bright category than the dim category. We have observed that a mere binary decision did not generate movements in a binary fashion unless the conceptual feature of the category could be reflected in the motor properties. The six different stimulus lengths significantly adjusted the action in a continuous manner in Experiment 2B. The ideomotor pathway (route 5 in Figure 1) might play a role in this effect. However, this experimental design is not strong enough to determine whether this adjustment is due to the ideomotor or perceptuo-motor effect. Thus, that question should be further explored using a new design.

## General discussion

The original 19th-century ideomotor theory considered how an action might be controlled by an inner source of thoughts without environmental stimulation. Prior to the recent revitalization of the term *ideomotor*, it was mainly used with apraxia, which is characterized by deficits in converting knowledge of action into actual behavior (Wheaton and Hallett, 2007). The ideomotor theory proposes that the idea of sensory consequences mediates between the intention to act and motor control. Accordingly, the key prediction of the theory is that actions can be generated solely by an idea without an accompanying stimulus or effect. However, until recently, the ideomotor theory has been examined using only variations in what constitutes an *idea* rather than by directly testing the critical prediction. Although the Theory of Event Coding suggests that the brain should construct a unified form for perception and control that could be represented in the brain in a distributed fashion (Hommel, 2004; Zmigrod and Hommel, 2013), that theory is based on evidence regarding motor activation *via* the effecto- or perceptuo-motor routes. In this study, we introduced a new experimental paradigm to demonstrate the essence of the original ideomotor hypothesis: An idea itself is sufficient to generate corresponding motor signals even when the conscious will or anticipation of achieving a consequence is absent. The form of *idea* required to control action is a highly abstract, amodal code that is not constrained by a specific sensory modality and can thus accommodate flexible multimodal representation. Our study has shown that an abstract category, deciding that a tone is “long,” generates motor code that induces a “long” movement.

When people make a decision about a non-spatial feature of a stimulus (e.g., blue or red) by pressing a spatially assigned key (e.g., left or right), the task-irrelevant spatial feature of the stimulus can facilitate or interrupt the response. Responses are faster in trials in which, e.g., the location of the stimulus and the response are compatible than in trials in which they are not. This is well known as Simon’s effect (Simon, 1969). The stimulus–response compatibility observed in Simon’s effects has been deemed supporting evidence for the Theory of Event Coding (Hommel, 2009). The task we devised here required participants to decide about a non-spatial feature of a stimulus (long or short in Experiments 1A, 1B, and 2A and bright or dim in Experiment 2B) by pressing a spatially assigned key (left or right). Contrary to Simon’s task, the stimulus property does not compete with the spatial response property at the selection stage. We used this task to observe how long participants would press the key according to their decision about the stimulus category. Participants recognized that response times and accuracy rates were being recorded because they received error messages when they made a mistake in those performances, but they were unaware that the response durations were being recorded. We found that their response durations were mainly modulated by their categorical thinking with marginal adjustment by their perception of the stimulus in Experiments 1B and 2A.

We also observed that the keypressing duration was modulated by the non-decisional property (i.e., stimulus duration) when participants categorized the stimulus brightness when both the duration and the brightness of the stimulus were varied with six levels in Experiment 2B. The decision about brightness, bright or dim, might have transferred its property to their movement such that the intensity of the brightness modulated the forcefulness of their action, but we did not measure that in this study. The observed stimulus duration effect could be explained by route 1 or route 2 in Figure 1. Route 1 indicates the activation of a certain idea by means of the perception of a stimulus, which eventually leads to the ideomotor route. Route 2 is affordance, which facilitates movement directly by the perception of an executable feature in the environment, without the bridge state of an idea. The stimulus duration itself that we used here is neither an executable nor a topographical property to act on, such as grasping a handle or keypressing a left key in response to a left-sided stimulus. Thus, we conjecture that the stimulus duration in Experiment 2B modulated the keypressing duration *via* route 1. Previous ideomotor theorists regarded an “ideo-” as an expectation of a resulting sensory effect, but our study shows that the idea is instead an abstract amodal attribute. Although the stimulus effect we found in Experiment 2B might be partly a function of the perceptuo-route, the ideomotor account (route 5) can explain both perceptual and ideational guidance more succinctly by assuming a single activation route.

Neuroscientific studies have found that the functional architectures of the action decision (what to do) and the motor specification (how to execute), which have traditionally been treated as separate domains, are topographically and temporally interconnected in the brain (Romo et al., 2004; Selen et al., 2012). In line with this research, the neural substrates for the execution and inhibition of action are also found to be overlapped at both the cortical (e.g., Coxon et al., 2006; Mirabella et al., 2011; Mattia et al., 2012) and subcortical levels (Mallet et al., 2016; Mancini et al., 2019). This interconnection can be demonstrated in diverse tasks, including natural interactions with objects in the world (e.g., catching, reaching, grasping, or executing saccadic eye movements; Favilla, 1997; Ghez et al., 1997; Selen et al., 2012) and computer-based tasks requiring arbitrary device control (Wallis and Miller, 2003). The interconnection could be a neural substrate for our finding that the decision category (what to do) is transferred to a movement feature (how to execute) unintentionally.

Procedural simplicity and low dependency on instruction allow this paradigm to be tested using animal action. Given that monkeys, mice, and pigeons can all learn to categorize stimuli (Penney et al., 2008), we might be able to determine whether the ideomotor route exists in non-human species by observing whether those animals press a lever longer when categorizing a stimulus as long. If so, that could provide evidence that non-human animals also have the ideomotor route for activating motor signals merely with their ideas rather than solely from habituation or stimulation.

Our findings allow us to reevaluate existing theories of perception and action. The Theory of Event Coding proposes that instigating an integrated assembly of event codes that share common features for perceived events and planned actions is the core mechanism of voluntary action (Hommel et al., 2001). As a modern variant of ideomotor theory, it proposes that both the stimulus and the image of action outcomes can activate common event codes to control action (for review, Shin et al., 2010). However, that theory is not specific enough to predict whether the stimulus property or cognitive representation of it modulates action control. The Theory of Event Coding has been criticized for its lack of power in constraining data (Hochberg, 2001) and is even considered non-falsifiable (Shin et al., 2010; Pfister, 2019). In terms of the Theory of Event Coding, this study has shown that the element of decision category, which is highly abstract, can provide a bridge state to bind perception and action. Our study can thus be used to elaborate ideomotor theory and build a testable hypothesis about the relationship between perception and action.

## Data availbility statement

The datasets presented in this study can be found in online repositories. The names of the repository/repositories and accession number(s) can be found at: https://osf.io/7mqkd/?view_only=32b4008ea2cc493ca63fb6e962475726.

## Ethics statement

The Institutional Review Board of Ulsan National Institute of Science and Technology approved this study (UNISTIRB-18-39-C). The patients/participants provided their written informed consent to participate in this study.

## Author contributions

YS and O-SK developed the study concept and the experimental design. YS, SC, and O-SK were performed testing and data collection. YS wrote the first draft. All authors performed the data analysis and interpretation, edited the manuscript, and approved the final version of the manuscript for submission.

## Funding

This work was supported by the Ministry of Education of Republic of Korea and the National Research Foundation of Korea (NRF-2022S1A5A8054398 to YS) and Ulsan National Institute of Science and Technology (UNIST) Research Grant 1.210046.01 to O-SK.

## Conflict of interest

The authors declare that the research was conducted in the absence of any commercial or financial relationships that could be construed as a potential conflict of interest.

## Publisher’s note

All claims expressed in this article are solely those of the authors and do not necessarily represent those of their affiliated organizations, or those of the publisher, the editors and the reviewers. Any product that may be evaluated in this article, or claim that may be made by its manufacturer, is not guaranteed or endorsed by the publisher.
